# Effect of Resin Acid and Zinc Oxide on Immune Status of Weaned Piglets Challenged With *E. coli* Lipopolysaccharide

**DOI:** 10.3389/fvets.2021.761742

**Published:** 2021-12-23

**Authors:** Xiaonan Guan, Regiane R. Santos, Hannele Kettunen, Juhani Vuorenmaa, Francesc Molist

**Affiliations:** ^1^Schothorst Feed Research, Lelystad, Netherlands; ^2^Hankkija Ltd, Hyvinkää, Finland

**Keywords:** weaning, stress, piglets, infection, zinc oxide, resin acid concentrate

## Abstract

With the ban of zinc oxide (ZnO) at high dosages in piglet diets in Europe by 2022, alternative nutritional solutions are being tested to support piglet immune defence during their weaning, the most critical and stressful moment of pig production. The present study evaluated the effect of zinc oxide (ZnO; 2,500 mg/kg diet) and resin acid concentrate (RAC; 200 mg/kg diet) on the immune defence of weaned piglets challenged with lipopolysaccharide (LPS). Piglets were challenged at days 7 and 21 post-weaning, and blood was sampled 1.5 and 3.0 h after each challenge to determine serum levels of pro- and anti-inflammatory cytokines. The levels of serum tumour necrosis factor alpha (TNF-α) and interleukin 8 (IL-8) increased at days 7 and 21, and those of IL-6 at day 21 when challenged piglets were fed a diet supplemented with ZnO. In challenged piglets fed with RAC, the serum levels of IL-1β, IL-6, IL-8, IL-10 and TNF-α were increased at days 7 and 21, except for that of IL-1β, which was not affected at day 21. The increased levels of these cytokines indicate the successful immune-modulatory effect of ZnO and RAC, which appears as a candidate to replace ZnO in weaned piglets' diets.

## Introduction

Weaning usually occurs 3 to 4 weeks after birth, which is a critical moment for piglets because they are suddenly exposed to several environmental, dietary and physiological stress conditions (1). These stresses on an immature digestive tract and immune system leads to post-weaning diarrhoea and reduced feed intake (2). Due to the abrupt lack of passive immunity acquired via maternal milk, these piglets are exposed to inflammatory processes (3). Moreover, active immunity has not yet fully established, so that immune support is warranted (4). Often, these piglets will experience dysbiosis and intestinal inflammation, factors that predispose them to gastrointestinal infections, which are linked to 17% of piglet mortality in Europe (5).

In the past, antibiotics were added to post-weaning diets to avoid post-weaning intestinal disturbances and to promote growth. However, this strategy was banned due to the risk of antimicrobial resistance, favouring the use of non-antimicrobial alternatives to support piglet health around weaning (6). One of the most effective nutritional strategies has been the supplementation of post-weaning diets with therapeutic levels of zinc oxide (ZnO) (7, 8). This essential trace element is often added to the piglet diet not only because of its antimicrobial properties but also because it improves body weight gain (BWG) and supports immunity (9). To achieve such effects, ZnO is generally added at doses ranging from 2,000 to 4,000 mg/kg diet (10, 11). The nutritional zinc requirement for piglets around weaning is lower (80–100 mg) (12) than that supplemented in the feed, resulting in Zn excretion in the faeces, which leads to environmental contamination. To avoid this, as of 2022 the supplementation of pig diets with therapeutic levels of ZnO will be banned in Europe (13). Hence, alternative feed additives should be able to support the intestinal integrity and immune system of piglets at weaning.

Among different feed additive alternatives, resin acid concentrates (RAC) appear promising because of their anti-inflammatory properties. Resin acids derived from coniferous trees are phytochemicals that have been used since ancient times in Asian and Scandinavian traditional human medicine for treating wounds (14, 15). Aguirre et al. (16) reported that supplementation of resin acids in broiler diets supported mucosal integrity via suppression of intestinal matrix metalloproteinase (MMP) activity, which degrades collagen fibres. In the same study, in-feed resin acids reduced duodenal inflammatory T-cell abundance. Supplementation of RAC in sow diets showed a beneficial effect on colostrum IgG levels (17). To test whether feed additives are able to support piglet health, immune system activation and growth, a model that can challenge the immune system is desirable. Among different challenge models, a lipopolysaccharide (LPS) challenge model in weaned piglets is chosen because of its elucidation of the integrated pathophysiology of infection and inflammation. The LPS molecule is present on the outer surface of all gramme-negative bacteria, is widely used as an immune stimulant and has been used to model bacterial infection in experimental farm animals (18).

In the organism, LPS can bind to many macromolecules, such as albumin and lipopolysaccharide binding protein (LBP) (19). The formed LBP-LPS complex is then transferred to the membrane-bound receptors or CD14, enabling interactions with Toll-like receptors (TLRs) on cell membranes (20). As a result, pro-inflammatory cytokines, including tumour necrosis factor alpha (TNF-α) and interleukins (ILs) IL-1β, IL-6, and IL-12 will be released via signalling through TLRs (21). Specifically, TNF-α is produced very early in inflammation followed by waves of IL-1β and then by IL-6. IL-10 is one of the most potent anti-inflammatory cytokines and is required for protection in many animal models of inflammation, and it has important roles in the regulation of gut homeostasis during host defence (22, 23). The association between IL-10 and inflammatory intestinal disease has been demonstrated in both humans and animal models (23). Cytokine and chemokine interactions are complex, but many previous studies only focused on two or three pro-inflammatory cytokines, including IL-1β, IL-6, and TNF-α (24), with or without the anti-inflammatory cytokine IL-10 (18). To properly interpret the effects of a dietary intervention on the inflammatory response, it is necessary to include other cytokines in the analysis, such as IL-8, IFN-α, IFN-γ, and IL-4. On this basis, the aim of the present study was to evaluate the effect of dietary resin acid concentrate (RAC) in comparison with ZnO on the immune responses of weaned piglets subjected to a LPS challenge.

## Materials and Methods

### Animals and Housing

In total, 48 healthy piglets, 24 females and 24 males (Tempo × Great Yorkshire and Landrace), with an average body weight of 7.5 ± 0.36 kg and an average age of 26 ± 0.9 days entered the experiment at the day of weaning. Before weaning, the piglets were fed creep feed from approximately day 7 of age, and immediately after weaning they were fed the experimental diets. A randomisation process was used to allocate piglets to replicates based on weaning weight. Piglets were housed in groups of six with half females and half males per pen (2.00 × 1.13 m) with partially slatted floors in climate-controlled units in the Schothorst Feed Research (SFR) weaner facility. Experimental diets were fed from weaning until 22 days post-weaning. The ambient temperature was gradually decreased from 29°C at the day of weaning to 22°C at 22 days post-weaning. Room temperature and relative humidity were recorded daily.

### Diets and Experimental Design

Diets were prepared as mash and were formulated free of antimicrobial growth promoters. The nutrient content of the diets met the requirement according to the NRC recommendation (12), as shown in Supplementary Table 1. RAC is a feed additive powder with 37.5% resin acids dried onto 62.5% of food grade carrier, produced using a standardised manufacturing procedure by Hankkija Oy (Hyvinkää, Finland). The 200 mg/kg dose of RAC thus provided 75 mg resin acids per kg feed. A basal diet was prepared and divided into three portions. The first portion became diet A and was regarded as the negative control diet (NC). To the second portion (basis for diet B), 2,500 mg ZnO/kg feed was added and it became diet B (i.e., NC + ZnO). To the third portion, 200 mg RAC/kg feed was added, and it became diet C (i.e., NC + RAC).

The experiment was performed in a completely randomised block design with four treatments and 12 replicates, where each piglet was an experimental unit, as described in Table 1. In brief, the 48 piglets used in the present study were housed in groups of 6 piglets (3 female and 3 male piglets) per pen. Piglets from treatment 1 were challenged with an intramuscular (i.m.) injection of phosphate buffered saline (PBS), and piglets from treatments 2 to 4 were challenged with an i.m. injection of LPS (L2630—LPS from *Escherichia coli* 0111:B4, Sigma-Aldrich®, Saint Louis, MI, USA) at a dose of 50 μg/kg live body weight. Piglets from treatments 1 and 2 were fed diet A and were regarded as treatment PBS + NC and treatment LPS + NC, respectively. Piglets from treatment 3 were fed diet B and consisted of treatment LPS + ZnO. Piglets from treatment 4 were fed diet C, and it became treatment LPS + RAC.

**Table 1:** Experimental treatments and diets^a^.

**Treatments**	**Description**	**Challenge**	**Experimental diet**
1	PBS + NC	PBS	Negative control (NC)
2	LPS + NC	LPS	NC
3	LPS + ZnO	LPS	NC + ZnO
4	LPS + RAC	LPS	NC + RAC

*ZnO, zinc oxide; RAC, resin acid concentrate*.

*a In total 48 piglets were used in the experiment with 2 pens per treatment and 6 piglets (3 female and 3 male piglets) per pen*.

### Challenge

On days 7 and 21 post-weaning, piglets from treatments 2 to 4 were challenged via i.m. injection with LPS. Piglets from treatment 1 were used as negative control and were treated in a similar way to the LPS-challenged group, but PBS was used. On days 7 and 21 post-weaning, blood was collected by venepuncture of the jugular vein at 1.5 and 3.0 h post i.m. injection of PBS or LPS. All the piglets were sampled. The sampling moments of 1.5 and 3.0 h post-challenge were chosen because a response to LPS infection reaches its peak between 1.0 and 3.0 h and declines slowly afterwards (25). An extra blood sample was obtained on day 14, when no PBS or LPS was administered to piglets. Blood was collected in 5-mL vacutainer tubes, centrifuged (3,000 *g* for 10 min), and the harvested serum was stored at −80°C until analysis. Faecal samples were collected the day after the challenge day at days 8 and 22 post-weaning and were stored at −80°C until analysis.

### Measurements

#### Performance and Health Status

Piglets were individually weighed at days 0 and 21 post-weaning. Feed intake per pen on day 21 post-weaning was recorded. Average daily gain (ADG) and the feed conversion ratio (FCR) were consequently calculated from days 0 to 21. Faecal consistency was visually recorded twice a week by an experienced panel on a scale from 1 (liquid faeces) to 8 (hard and dry faeces). Faecal score 6 was considered the optimal faecal score. To monitor the health status of all piglets, rectal temperature was measured at 6 h after the LPS injection on days 7 and 21, and on day 8. All piglets were observed daily for abnormal behaviour and clinical signs of illness. Medical treatments were registered.

#### Serum Analysis

Serum levels of IL-10, IL-1β, IL-6, TNF-α, IL-12, IL-8, IFN-α, IFN-γ, and IL-4 were analysed using the Luminex × MAP technology, a multiplexed sandwich immunoassay (Thermo Fisher Scientific Inc., Waltham, USA). The measurements were determined using the ProcartPlex Porcine Cytokine and Chemokine Panel 1 (catalogue number EPX090-60829-901) according to the manufacturer's instructions (Thermo Fisher Scientific Inc., Waltham, USA). The concentrations of the different cytokines were expressed as pg/mL and calculated according to a standard curve. The limit of detection level was set as 95% of the minimum detected value within the standard range. The limits of detection for IL-10, IL-1β, IL-6, IL-8, TNF-α, IL-12, IFN-α, IFN-γ, and IL-4 were 22.09, 4.03, 4.6, 10.96, 19.02, 11.36, 0.47, 38.01, and 0.54 pg/mL, respectively.

#### Faecal Analysis

Briefly, faeces were weighed and triturated in hexadecyltrimethylammonium bromide buffer solution (HTAB, Sigma-Aldrich, ST. Louis, USA) and centrifuged (1,000 g, 15 min, at 4°C) to obtain the faecal extract. Then, total peroxidase (TPO) activity was evaluated by a modified method of Bradley et al. (26) based on oxidation of o-dianisidine by hydrogen peroxide, with or without addition of faecal extract. The results were reported as TPO units/g faeces). Limit of quantification = 0.56 U/mg faeces.

#### Statistical Analysis

Statistical analysis was carried out with GenStat® for Windows (20th edition; VSN International, Hemel Hempstead, UK). All parameters were analysed by ANOVA with Fisher's least significant difference (LSD) to compare treatment means. *Post-hoc* multiple comparisons between treatment groups were tested via Tukey's test. For serum analysis the piglet was the experimental unit, classification factors included in the model for serum parameters were the treatment (1, 2, 3, and 4), day of challenge (7 and 21), time post-challenge (1.5 and 3.0 h) and their interactions (treatment × day of challenge × time post-challenge). Classification factors included in the model for performance and faecal score was the treatment (1, 2, 3, and 4), where the pen was the experimental unit. Values with *P* ≤ 0.05 were considered statistically significant.

## Results

### General Observations

All piglets were healthy prior to LPS challenge. After the LPS challenge, piglets developed signs including vomiting, loss of activity and lethary. All the piglets recovered to normal within 24 h after the LPS challenge.

The growth performance at pen level is presented in Table 2. No differences in body weight were observed among experimental treatments. Likewise, no differences in performance were observed in the overall experimental period. The faecal score was similar among experimental treatments, except that the LPS-challenged piglets fed with the ZnO diet showed dryer faeces.

**Table 2:** Effect of experimental treatments on piglet performance^1^.

	**PBS+NC**	**LPS+NC**	**LPS+ZnO**	**LPS+RAC**	***P*-value**	**LSD^2^**
***BW (kg)***						
Day 0	7.5	7.5	7.5	7.5	0.26	0.02
Day 21	11.7	11.1	13.2	11.2	0.29	3.03
***Day 0–21***						
ADG (g)	198.9	167.9	267.6	174.3	0.29	144.8
ADFI (g)	343.1	306.2	385.0	292.5	0.35	146.0
FCR (g:g)	1.724	1.920	1.440	1.702	0.38	0.7230
FS (score)^3^	5.5^a^	5.7^a^^b^	6.1^b^	5.5^a^	0.04	0.43

a,b * Different superscripts indicate significant differences among treatments (P ≤ 0.05)*.

1 *In total 48 piglets were used in the experiment with 2 pens per treatment and 6 piglets (3 female and 3 male piglets) per pen*.

2 *LSD is least significant difference at α < 0.05*.

3 *Faecal score was registered on a scale from 1 to 8 (1 = liquid faeces, 6 = optimal faeces, 8 = hard dry faeces) and was scored twice a week on a pen level*.

*BW, body weight; ADG, average daily gain; ADFI, average daily feed intake; FCR, feed conversion ratio; FS, faecal score*.

**Figure 1:**
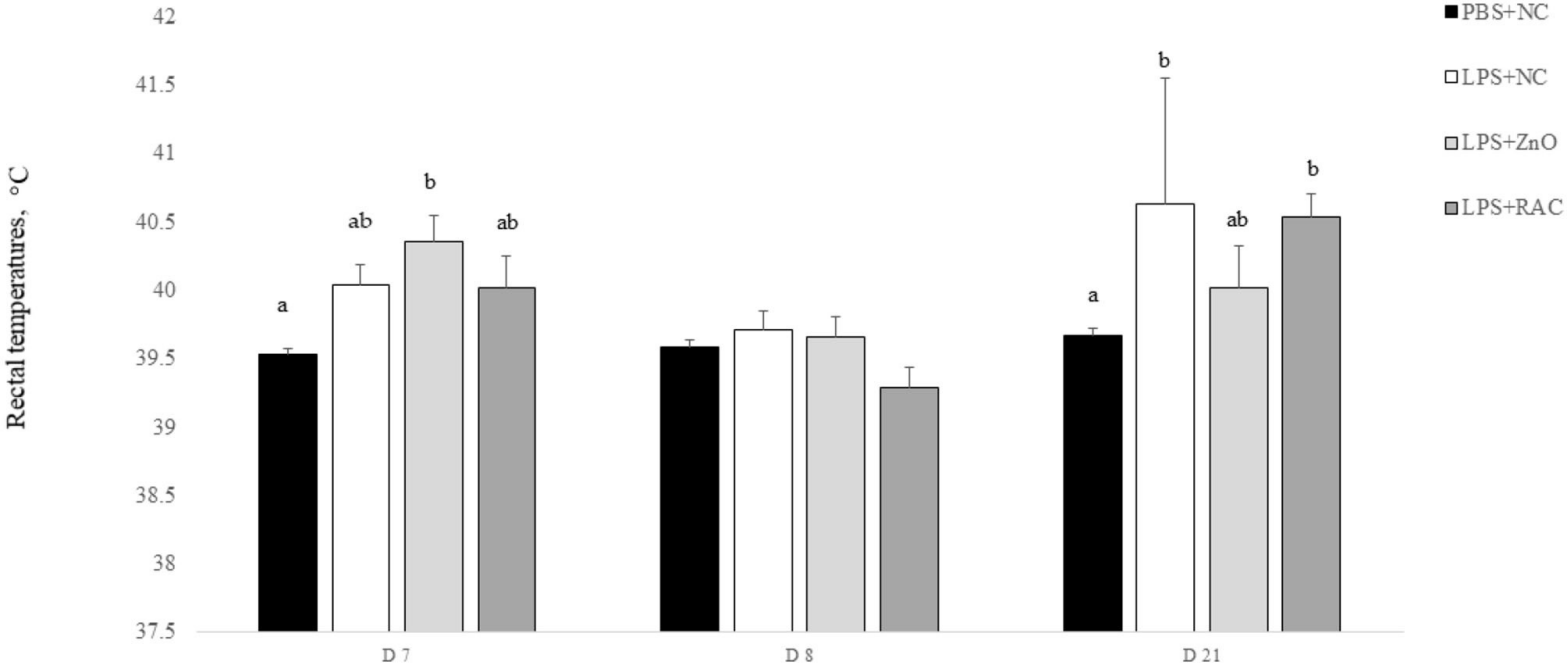
Mean (±SEM) rectal temperature of piglets from the PBS+NC, LPS+NC, LPS + ZnO, and LPS + RAC groups. ^a,b^ Different superscripts indicate significant differences among treatments per sampling day (*P* ≤ 0.05)^1^. ^1^Rectal temperature was measured in all piglets at 6 h after the LPS injection on days 7 and 21, and on day 8.

The rectal temperature of individual piglets is presented in Figure 1. On day 7, the rectal temperature of piglets who received the LPS challenge increased substantially compared with that of piglets who only received PBS (*P* = 0.006). On day 8, there were no differences in rectal temprature across the treaments. On day 21, piglets from treatments LPS + NC and LPS + RAC had the highest rectal temprature, followed by piglets from the treatment LPS + ZnO, and piglets from treament PBS + NC showed the lowest rectal temprautre (*P* = 0.002).

### Serum Cytokine Profile

At day 14, all the assessed cytokines were at basal levels. Therefore, statistical analysis considered data from days 7 and 21, at both time points, i.e., 1.5 and 3.0 h post-challenge. Serum levels of IL-4, IFN-α, and IFN-γ were below the limit of detection. Serum level of IL-1β was below the limit of detection for 80% of the piglets in all the treatments.

**Figure 2:**
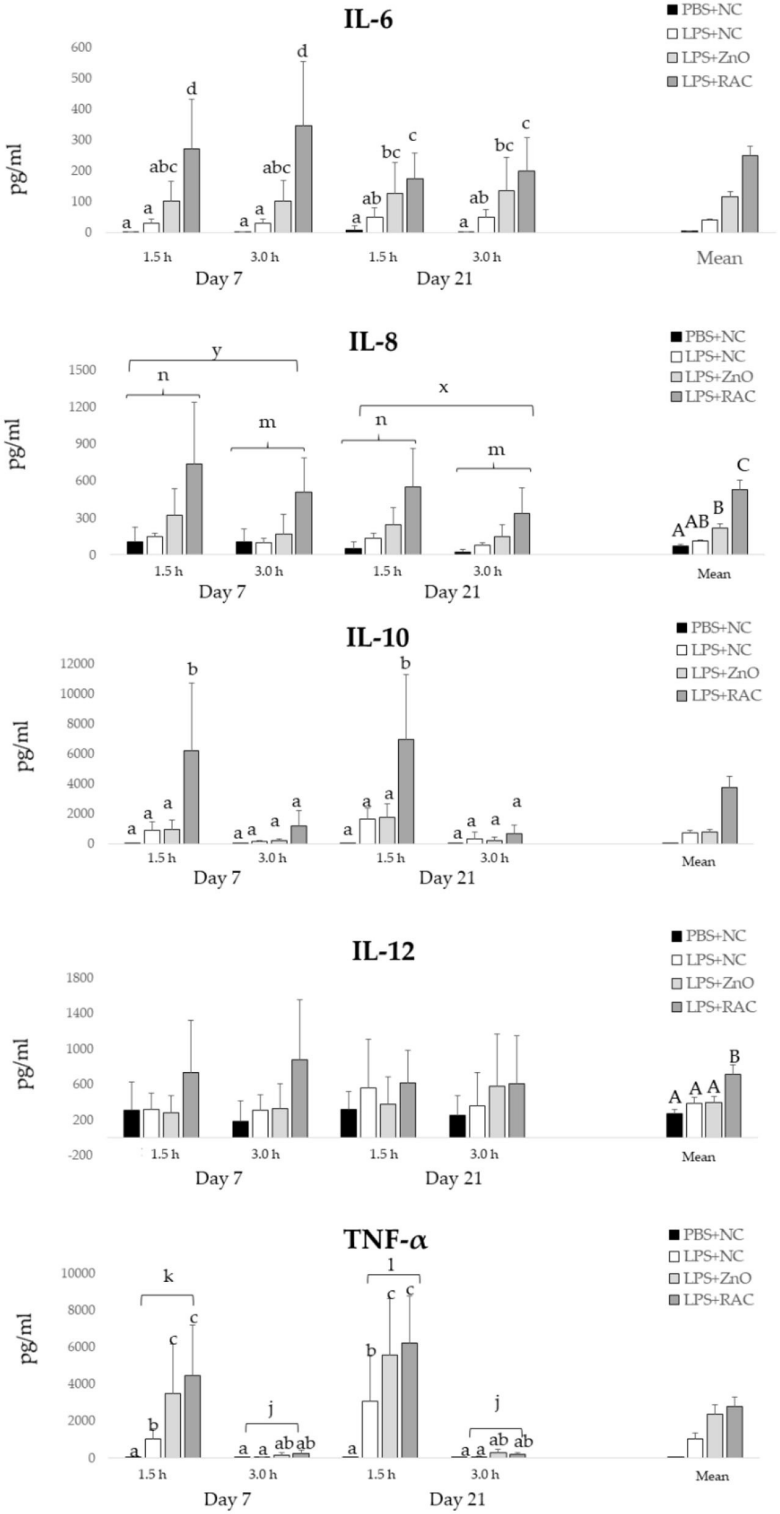
Mean (±SEM) serum levels of IL-6, IL-8, IL-10, IL-12, and TNF-α in piglets from the PBS+NC, LPS+NC, LPS + ZnO, and LPS + RAC groups. ^a−d^ Different superscripts indicate significant differences among treatments and day or sampling time (*P* ≤ 0.05), ^A−C^ Different superscripts indicate significant differences among treatments (*P* ≤ 0.05), ^x,y^ Different superscripts indicate significant differences among days (*P* ≤ 0.05), ^m,n^ Different superscripts indicate significant differences among sampling time (*P* ≤ 0.05), ^j−l^ Different superscripts indicate significant differences among day and sampling time (*P* ≤ 0.05)^1^. ^1^Blood samples were collected from all piglets (*n* = 48) on days 7 and 21 post-weaning, and at 1.5 and 3.0 h post i.m. injection of PBS or LPS.

Figure 2 depicts the serum levels of IL-6, IL-8, IL-10, IL-12, and TNF-α. A significant interaction between treatments and days on the IL-6 level was observed (*P* = 0.004). At day 7 and day 21, IL-6 serum levels were significantly higher in piglets from treatments LPS + ZnO and LPS + RAC compared with piglets from treatments PBS + NC and LPS + NC. At day 7, piglets from treatment LPS + RAC showed higher serum IL-6 than piglets from treatment LPS + ZnO. However, at day 21, piglets from treatments LPS + RAC and LPS + ZnO showed similar serum IL-6 level. For IL-8, a significant effect of the experimental treatment (*P* < 0.001), day (*P* = 0.005) and time (*P* = 0.032) were observed. Piglets from treatment LPS + RAC showed the highest levels of serum IL-8, followed by piglets from LPS + ZnO, and piglets from PBS + NC and LPS + NC showed the lowest serum IL-8 levels. IL-8 levels were higher at day 7 than day 21, and were higher at 1.5 h than 3 h after sampling. A significant interaction between the experimental treatment and sampling time was observed on the IL-10 serum level (*P* < 0.001), and no day effect was observed. Serum levels of IL-10 were significantly increased only in LPS-challenged piglets fed RAC diets sampled 1.5 h after the challenge. The serum IL-12 level was significantly higher in piglets from treatment LPS + RAC than in the other treatments (*P* < 0.001). For TNF-α, there were significant interactions between the treatment and sampling time (*P* < 0.001), and between the day and sampling time (*P* = 0.020). At day 7, TNF-α serum levels were significantly increased in challenged piglets fed the RAC or ZnO diets, and sampled 1.5 h after challenge. Similar effects were observed at day 21, where challenged piglets fed the NC diet also presented increased TNF-α serum levels, but significantly lower than those piglets from treatments LPS + ZnO and LPS + RAC.

### Faecal TPO Activity

TPO activity in the faeces are presented in Supplementary Figure 1. No treatment or day effect was found. On day 8, the faecal TPO activity were 5.4, 4.6, 4.7, and 4.7 U/mL for treatments PBS+NC, LPS+NC, LPS+ZnO, and LPS+RAC, respectively. On day 22, the faecal TPO activity was numerically the greatest in treatment LPS+NC (7.4 U/mL), and it was 5.6, 5.9, and 6.3 U/mL for treatments PBS+NC, LPS+ZnO, and LPS+RAC, respectively.

## Discussion

In the present study, we demonstrated an immune-modulatory effect when LPS challenged piglets were fed diets containing ZnO or RAC. All the piglets were obtained from the same commercial farm, with the same genetics, and were randomised based on litter, weaning body weight and gender, to guarantee a similar health and immune background. Moreover, they were housed in the same room and the environment was similar, supporting that the present data resulted from dietary intervention.

Although the performance analysis was used as a secondary and indicative parameter only, the similar feed intake and growth regardless of the treatments showed that the present challenge was mild. In general, it is accepted that activation of the immune system will require extra resources, otherwise the growth performance of piglets might be negatively affected. Numerically, piglets fed with LPS + ZnO apparently have a better performance in comparison with those fed with other diets. It is important to bear in mind that this trial was performed with a limited number of piglets to evaluate the effect of treatments on animal performance, and these data are only preliminary. To determine the dietary effects on growth, a larger number of replicates per treatment should be used. So, the main objective of this LPS challenge was to analyse the effect of different diets on immune system activation at systemic level. Increased faecal TPO activity indicates gut inflammation in weaned piglets (27). Only a numerically increased TPO activity was found in piglets challenged with LPS at the end of the study, suggesting that the current LPS challenge was not able to cause immediate inflammation on the gut level.

Both ZnO and RAC present immunomodulatory activity, as previously demonstrated. *In vivo* studies showed that dietary ZnO decreased intestinal inflammation in piglets by directly interfering with the expression of cytokines (28). Moreover, ZnO not only modulates cytokines, but acts in the development and function of the immune system (9, 29). Recently, the immunomodulatory properties of RAC was reported, by modulating gut microbiota and reducing inflammatory biomarkers (30). A direct anti-inflammatory effect of resin acid was demonstrated in mice lungs submitted to allergic inflammation (31), as well as by acting as a ligand for PPARs in macrophages and adipocytes (32). In-feed resin acids have inhibited the inflammation-associated collagen breakdown in the jejunum and ileum, and reduced the numbers of inflammatory T-cells in the duodenum of broiler chickens (16).

At 6 h after the LPS challenge on days 7 and 21, piglets that received the LPS challenge showed fever, and their rectal temperatures were above 40°C. The observed values are similar to those found in other studies using LPS as an immune system stimulator in pigs (33, 34). Within 24 h, the rectal temperature recovered, consistent with a previous study (34). The cytokine production profile after the LPS challenge was time dependent. Previous studies found that the peak level of the pro-inflammatory cytokine TNF-α occurred at 1 h after the LPS challenge, decreasing to pre-challenge levels at ~3 h. Another pro-inflammatory cytokine, IL-6, peaked ~3 h after the LPS challenge and subsequently fell at ~6 h (34, 35). The current study demonstrated a consistent pattern of TNF-α and IL-6 production in response to the LPS challenge. Stimulation of the innate immune system activates the production of pro-inflammatory cytokines. Activated macrophages, monocytes, neutrophils, and other cells are able to produce TNF-α, which initiates the inflammatory response but also limits the extent and duration of the inflammatory process (36). Such a balance is confirmed by the concomitant production of the anti-inflammatory cytokine IL-10, which is also produced by macrophages, monocytes and B and T cells. Alterations in the balance of TNF-α and IL-10 production will affect macrophage function and cell survival (37).

Among LPS-challenged piglets, supplementation of ZnO at 2,500 mg/kg diet or with RAC at 200 mg/kg stimulated TNF-α at days 7 and 21 (1.5 h post-challenge) and the production of IL-8 and IL-6, regardless of challenge day and sampling time. The effect of RAC supplementation on IL-6 and IL-8 was stronger than that of ZnO supplementation, but similar effects on TNF-α were found. Interestingly, among LPS-challenged piglets, IL-10 production was only increased in piglets fed the RAC diet at days 7 and 21 (1.5 h post-challenge). The increased levels of these cytokines indicate the successful immune modulatory effect of both ZnO and RAC, with some differences between the two. Both TNF-α and IL-10 were stimulated in the first 1.5 h post-challenge. Given the fact that the challenged piglets fed supplemented diets were not more ill than those fed the control diet and no differences in animal performance were observed, it can be suggested that both ZnO and RAC supported this pro- and anti-inflammatory balance. Notably, ZnO did not stimulate higher IL-10 production than the control diet. Increased IL-10 has been related to improved intestinal barrier function (38). However, one must bear in mind that the present study evaluated systemic response.

Scheller et al. (39) demonstrated that although IL-6 is a pro-inflammatory cytokine, it also has regenerative activities that, if absent, would result in a more aggravated inflammatory process. Piglets fed RAC-supplemented diets showed an increase in IL-6 serum levels, followed by those fed ZnO, indicating that immune modulation was taking place during the challenge and that probably the RAC were able to counteract the LPS challenge as well as support tissue regeneration. Regarding IL-8, also called chemokine CXCL8, this cytokine is particularly important in aiding migration of additional neutrophils to inflammatory sites (40). Challenged piglets fed a diet supplemented with RAC showed the strongest IL-8 activation, followed by piglets fed the ZnO diet. Again, this is an indication that RAC has a robust immune modulatory effect in LPS-challenged piglets.

## Conclusions

In conclusion, an immune-modulatory effect is observed in LPS-challenged piglets fed a diet supplemented with 2,500 mg ZnO/kg or 200 mg RAC/kg. Based on the serum cytokine profile analysis, the stimulation of anti- and pro-inflammatory cytokines was more pronounced in piglets fed a diet supplemented with 200 mg RAC/kg than in those fed the diet supplemented with 2,500 mg ZnO/kg.

## Data Availability Statement

The original contributions presented in the study are included in the article/supplementary material, further inquiries can be directed to the corresponding author/s.

## Ethics Statement

The study was conducted according to the restrictions of the Animal and Human Welfare Codes in The Netherlands, and approved by the Ethics Committee and Institutional Review Board of Schothorst Feed Research (AVD246002015279, June 3rd, 2019).

## Author Contributions

XG and FM: conceptualization and funding acquisition. XG, HK, JV, and FM: methodology. XG, RS, and FM: validation and writing—original draft preparation. XG and RS: formal analysis. XG: investigation and project administration. FM, HK, and JV: resources. XG, RS, HK, JV, and FM: writing—review and editing. All authors have read and agreed to the published version of the manuscript.

## Conflict of Interest

HK and JV were employed by company Hankkija Ltd. The remaining authors declare that the research was conducted in the absence of any commercial or financial relationships that could be construed as a potential conflict of interest.

## Publisher's Note

All claims expressed in this article are solely those of the authors and do not necessarily represent those of their affiliated organizations, or those of the publisher, the editors and the reviewers. Any product that may be evaluated in this article, or claim that may be made by its manufacturer, is not guaranteed or endorsed by the publisher.
