# Supplementary material for: Effect of Resin Acid and Zinc Oxide on Immune Status of Weaned Piglets Challenged With E. coli Lipopolysaccharide
Source: Front Vet Sci. 2021 Dec 23;8:761742. doi: 10.3389/fvets.2021.761742 (PMC8733644; doi:10.3389/fvets.2021.761742)
Supplement: Supplementary file 1 [file Data_Sheet_1.pdf]

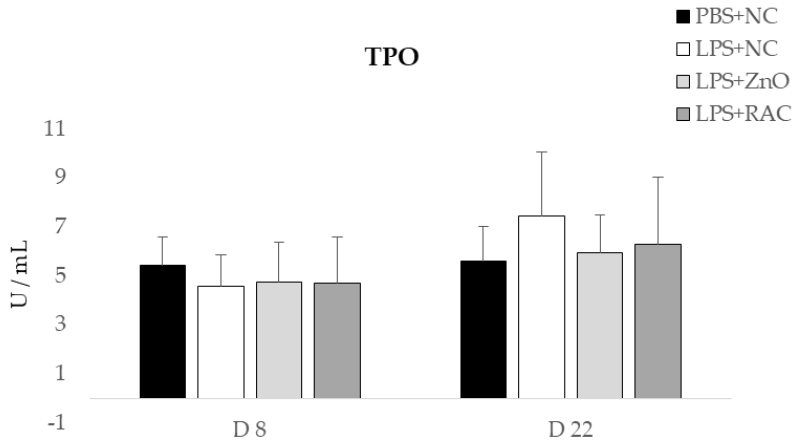

Supplementary Figure 1. Total peroxidase (TPO) activity (units/g faeces) of piglets from the PBS+NC, LPS+NC, LPS + ZnO and LPS + RAC groups.[1]

[1] Faecal samples were collected from all the piglets (n=48) at days 8 and 22 (i.e. the day after the challenge).

Supplementary Table 1. Composition (%) and calculated nutrient levels (g/kg) of the experimental diets.

**Ingredients**	**Units**	**Diet A**	**Diet B**	**Diet C**
Potato protein	%	3.23	3.23	3.23
Barley	%	25.00	25.00	25.00
Maize	%	30.00	30.00	30.00
Maize gluten ml 60% CP	%	5.00	5.00	5.00
Molasses cane>47.5% su	%	1.00	1.00	1.00
Soybean oil	%	1.00	1.00	1.00
Soya bean meal47% CP	%	7.50	7.50	7.50
Wheat	%	9.64	9.64	9.64
Monocalcium phosphate	%	0.34	0.34	0.34
Whey p.delac 18% ash	%	11.81	11.81	11.81
Salt	%	0.50	0.50	0.50
Sunflower seed meal 38% CP	%	2.00	2.00	2.00
Lysine-HCl (L 79%)	%	0.21	0.21	0.21
Methionine (DL 99%)	%	0.05	0.05	0.05
Threonine (L 98%)	%	0.02	0.02	0.02
Lys+trypt premix	%	0.77	0.77	0.77
Limestone	%	1.32	1.32	1.32
Premix	%	0.60	0.60	0.60
Zinc oxide (ZnO)	mg/kg	-	2500	-
Resin acid concentrate (RAC)*	mg/kg	-	-	200
**Calculated nutrients (g/kg)**				
Net energy	MJ/kg	9.86	9.86	9.86
Dry matter	g/kg	939	939	939
Ash	g/Kg	60.85	60.85	60.85
Crude protein	g/Kg	189.28	189.28	189.28
Crude fibre	g/Kg	23.42	23.42	23.42
Sugar	g/Kg	78.73	78.73	78.73
Starch	g/Kg	385.31	385.31	385.31
SID_LYSs	g/Kg	11.12	11.12	11.12
SID_METs	g/Kg	3.65	3.65	3.65
SID_M+Cs	g/Kg	6.53	6.53	6.53
SID_THRs	g/Kg	7.00	7.00	7.00
SID_TRPs	g/Kg	2.16	2.16	2.16
SID_ARGs	g/Kg	8.18	8.18	8.18
SID_ILEs	g/Kg	7.23	7.23	7.23
SID_VALs	g/Kg	8.26	8.26	8.26
SID_HISs	g/Kg	3.83	3.83	3.83
SID_ALAs	g/Kg	8.75	8.75	8.75
SID_ASPs	g/Kg	14.19	14.19	14.19
SID_GLUs	g/Kg	31.11	31.11	31.11
SID_GLYs	g/Kg	5.72	5.72	5.72
SID_LEUs	g/Kg	16.63	16.63	16.63
SID_PHEs	g/Kg	8.42	8.42	8.42
SID_PROs	g/Kg	12.19	12.19	12.19
SID_SERs	g/Kg	8.18	8.18	8.18

SID_TYRs	g/Kg	6.40	6.40	6.40

* RAC is a feed additive powder with 37.5% resin acids dried onto 62.5% of food grade whole grain wheat flour, produced by Hankkija Oy (Hyvinkää, Finland).
